# Canned Meat and Typhoid Fever

**Published:** 1899-02

**Authors:** 


Canned Meat and Typhoid Fever.—It has been seriously charged by certain persons through the public press that the canned meats issued to the troops was, or might have been, the cause of the typhoid fever in the army. For myself, I dissent in toto from the proposition that canned meats and soups may, or can cause typhoid fever. It is not in accord with the accepted teachings on the subject. I do not believe a single well-informed physician in the world will endorse it, or that a single recognized authority can be cited in support of it. I deny that canned meats or soups can, by any possibility, produce typhoid fever. Every physician knows that typhoid fever is caused by a specific living micro-organism,—technically, the *bacillus typhoides* (the bacillus of Eberth),—a product of filth, which is usually communicated to the human system through the medium of polluted drinking water. Canned meats and soups cannot, by any possibility, generate this bacillus, and typhoid fever can be caused by nothing else whatever. It would be as reasonable to say that smallpox, yellow fever, or diphtheria or scarlet fever, can be produced by canned meats or soups,—diseases which, like typhoid fever, have their own specific micro-organism, and can be caused by nothing else. Canned meats and soups are sterilized by boiling, and are hermetically sealed at or beyond the boiling point. So long as the air is excluded, they will keep indefinitely, with ordinary care, and will furnish wholesome, nutritious food.* Should the can be defective, or be not perfectly sealed; should a particle of air enter the can, fermentation will take place, the can will swell, and thus show that the contents have *become unfit for use.* If, however, a portion of the contents should be eaten, the result will be *acute poisoning;* vomiting, purging, prostration, and, if not relieved, collapse and speedy death; a matter of a few hours. This poisoning is produced by a "toxine," or "ptomaine" (alkaloidal), generated by bacterial action, a poison bearing no more analogy to the typhoid bacillus than a fish bears to a stone; the one being organic, the other, inorganic.

By the bye, in this connection it is well enough to call attention to the fact that typhoid fever was the scourge of both armies during the civil war, and the Confederate soldiers, at least, were not fed on canned meats and soups.

*Canned foods are said to have been found at Pompeii, sound, after eighteen hundred years.

